# From museum drawer to tree: Historical DNA phylogenomics clarifies the systematics of rare dung beetles (Coleoptera: Scarabaeinae) from museum collections

**DOI:** 10.1371/journal.pone.0309596

**Published:** 2024-12-31

**Authors:** Fernando Lopes, Nicole Gunter, Conrad P. D. T. Gillett, Giulio Montanaro, Michele Rossini, Federica Losacco, Gimo M. Daniel, Nicolas Straube, Sergei Tarasov

**Affiliations:** 1 Finnish Museum of Natural History, University of Helsinki, Helsinki, Uusima, Finland; 2 Biodiversity and Geosciences Program, Queensland Museum Kurilpa, Brisbane, Queensland, Australia; 3 Department of Agronomy, Food, Natural Resources, Animals and Environment (DAFNAE), University of Padova, Veneto Region, Padua, Italy; 4 Department of Terrestrial Invertebrates, National Museum Bloemfontein, Bloemfontein, Free State province, South Africa; 5 Department of Biological and Environmental Sciences, Walter Sisulu University, Mthatha, South Africa; 6 Department of Natural History, University Museum of Bergen, Vestland, Norway; Central University of Punjab, INDIA

## Abstract

Although several methods exist for extracting and sequencing historical DNA originating from dry-preserved insect specimens deposited in natural history museums, no consensus exists as to what is the optimal approach. We demonstrate that a customized, low-cost archival DNA extraction protocol (∼€10 per sample), in combination with Ultraconserved Elements (UCEs), is an effective tool for insect phylogenomic studies. We successfully tested our approach by sequencing DNA from scarab dung beetles preserved in both wet and dry collections, including unique primary type and rare historical specimens from internationally important natural history museums in London, Paris and Helsinki. The focal specimens comprised of enigmatic dung beetle genera (*Nesosisyphus*, *Onychothecus* and *Helictopleurus*) and varied in age and preservation. The oldest specimen, the holotype of the now possibly extinct Mauritian endemic *Nesosisyphus* *rotundatus*, was collected in 1944. We obtained high-quality DNA from all studied specimens to enable the generation of a UCE-based dataset that revealed an insightful and well-supported phylogenetic tree of dung beetles. The resulting phylogeny propounded the reclassification of *Onychothecus* (previously *incertae sedis*) within the tribe Coprini. Our approach demonstrates the feasibility and effectiveness of combining DNA data from historic and recent museum specimens to provide novel insights. The proposed archival DNA protocol is available at DOI 10.17504/protocols.io.81wgbybqyvpk/v3.

## Introduction

Museomics, a term encompassing procedures allowing access to and analysis of the historical genomic data preserved in biological specimens deposited in natural history museums, is providing unprecedented opportunities to investigate evolutionary histories [1, 2]. Together with concomitant advances in high-throughput sequencing technologies and bioinformatics, museomics has paved the way for the exploitation of an ever-broader diversity of taxonomic and temporal sampling [3, 4]. Importantly, by enabling access to genomes already preserved in existing museum specimens, museomics can circumvent the need for costly, laborious and unpredictable bespoke fieldwork, to achieve taxon sampling objectives [2, 3]. Museomics is also compatible with physically preserving the morphological integrity of specimens when non-destructive DNA extraction methods are employed. This is of paramount importance to natural history museums and the scientific community because it ensures that intact voucher specimens will remain available for study by future generations [5, 6]. Indeed, the importance of museomics can only heighten as the necessity for inclusion of recently extinct species within phylogenies becomes increasingly inevitable [7]. Progress in insect museomics has already greatly contributed to the study of insects—Earth’s most diverse organisms [5, 8]. Although notable recent achievements in DNA extraction mean that the recovery of DNA from dry-pinned museum specimens is no longer remarkable [9], challenges still remain [2]. Specifically, the DNA in many dry-preserved museum specimens is fragmented and prone to contamination, whilst the comparatively small amount of tissue present in small insects can further limit the success of DNA extractions [10].

In recent years, a variety of molecular methods have been developed to obtain historical DNA data at a genome-wide scale [3, 4], including approaches exploiting both whole-genome (shotgun) and reduced representation sequencing [7, 11, 12]. Many widely used methods rely on standard DNA extractions using commercial DNA kits, followed by the construction of DNA libraries based on hybridization capture approaches that combine restriction enzyme fragmentation and RNA probe capture. For instance, hyRAD uses a double enzymatic restriction of DNA extracts from fresh samples (containing well-preserved DNA) to produce RNA probes that serve as baits for capturing homologous fragments from historical (more degraded) DNA libraries [13, 14]. However, standard DNA extraction, typically undertaken with commercially available kits, is optimized for high molecular weight DNA, only ineffectively capturing lower-weight short fragments, which are precisely those expected from degraded historical samples. Furthermore, reduced representation approaches exploiting restriction enzymes require a comparatively large initial amount of source DNA, not easily obtained from small insect specimens [15]. Moreover, those methods tend to be costly when extensively sampling a wide range of insect taxa. They are also labor-intensive because they require the creation of custom RNA probes for each taxon being studied. Crucially, such methods are susceptible to the drawbacks associated with restriction enzymes. These include the potential for enzyme mismatch, either due to point mutations because target taxa are too distantly related or due to DNA fragmentation at restriction sites (especially in poorly preserved samples); both processes that can lead to missing data [16].

Within museomics studies, more cost-effective genome reduction methods, such as Ultra Conserved Elements (UCEs) and Anchored Hybrid Enrichment (AHE) are rising in popularity due to their ability to target specific informative loci within a focal group [17–20]. While standard extraction from dry-preserved specimens may yield adequate DNA for UCE and AHE sequencing [20, 21], its success varies based upon specimen preservation. Therefore, exploring the effectiveness of more sensitive DNA extraction methods is essential, especially since their application in entomological collections remains poorly investigated [18].

In this study, we aim to bridge this gap by assessing a cost-effective (∼ €10 per sample) archival DNA extraction protocol [22] specifically tailored to historical insect specimens and downstream UCE sequencing. We applied this protocol, in combination with standard DNA extraction from fresh specimens, in addition to compiling relevant sequences deposited in GenBank, to explore the phylogenetic relationships of eleven species and subspecies of dung beetles (Coleoptera: Scarabaeinae) represented by historical specimens from three museums: The Natural History Museum, London (NHMUK); the Muséum National d’Histoire Naturelle, Paris (MNHN); and the Finnish Museum of Natural History, Helsinki (MZHF). We selected to focus our study on scarab dung beetles because they are of considerable biological interest, for providing important ecosystem services including nutrient cycling and secondary seed dispersal [23], in addition to having proven to be a dependable ‘proxy’ bioindicator taxon indicative of wider biodiversity patterns [24–27]. Hence, robustly infering their systematics is fundamental to accurate interpretation of their wider ecological significance.

The selected specimens are of diverse ages and represent enigmatic species of questionable phylogenetic assignment. The oldest specimen, the holotype of *Nesosisyphus rotundatus* Vinson, 1946 collected in 1944, and deposited in NHMUK, is a potentially extinct species from Mauritius, not previously included in molecular phylogenies (*e.g.*, Tarasov & Dimitrov (2016) [28]). The extremely rare (*i.e.* apparently infrequently collected and represented only by very few museum specimens) Oriental genus *Onychothecus* Boucomont, of uncertain taxonomic affinity [28] and hitherto lacking DNA data, was represented by a specimen collected in 1985 that is held in MNHN. Finally, nine poorly-known taxa belonging to the endemic Madagascan genus *Helictopleurus* D’Orbigny were represented by specimens collected between 2003–2010 and deposited in MZHF.

Our archival DNA extraction protocol yielded DNA of sufficiently high quality for successful UCE sequencing using the recently designed probe set for scarab beetles [29]. To elucidate the phylogenetic position of the selected enigmatic species, we expanded our taxon sampling to include additional dung beetle species represented by alcohol-preserved specimens, extracted using a standard commercial DNA extraction kit protocol. In the following sections, we discuss the phylogenetic position of the focal species based on our results and implement necessary taxonomic changes. We also explore the broader application of the proposed extraction protocol to a wide range of historical specimens of insects and other taxa.

## Materials and methods

This research did not involve human participants or live animals, so ethical approval was not required. The study followed the guidelines of the Madagascar Institut pour la Conservation des Ecosystèmes Tropicaux (MICET), the Mauritian National Parks and Conservation Service (NPCS), the Finnish Museum of Natural History Research Programme in Systematics and Evolution, and all other contributing institutions, ensuring adherence to ethical standards in scientific research.

### Taxon sampling

We compiled a dataset of UCE sequences from 96 beetles (S1 Table), encompassing mostly scarabaeoid beetle lineages from various biogeographical regions. Our dataset combined 70 newly-sequenced specimens for this study with existing data for 26 specimens from a previous study available on GenBank [29]. The ingroup consisted of 67 samples belonging to 42 genera or subgenera of true dung beetles of the subfamily Scarabaeinae. The outgroup consisted of 29 samples (of 26 genera) belonging either to scarab beetle families and subfamilies other than Scarabaeinae, or to non-scarab beetles (two species of Silphidae). Fifty-nine of the newly sequenced samples (representing 40 genera) originated from frozen (-20°C) alcohol-preserved “wet collection” specimens that were sourced from five natural history museums. A further 11 historical samples belonging to three genera (*Nesosisyphus* Vinson, 1946, *Onychothecus* and *Helictopleurus*) were selected from the dry collections of three museums, and formed the focal taxa for our study (Table 1).

**Table 1:** Dry-preserved scarab dung beetle specimens from natural history museum collections, used in historical DNA extractions. Natural History Museum, London (NHMUK); Muséum National d’Histoire Naturelle, Paris (MNHN); and Finnish Museum of Natural History (MZHF).

Sampling	Year of Sampling	Museum	Origin
*Helictopleurus fasciolatus fasciolatus* (Fairmaire, 1898)	2003	MZHF	Madagascar
*Helictopleurus fasciolatus obscurus* Lebis, 1960	2009	MZHF	Madagascar
*Helictopleurus fasciolatus pseudofasciolatus* Montreuil, 2007	2010	MZHF	Madagascar
*Helictopleurus neuter* (Fairmaire, 1898)	2009	MZHF	Madagascar
*Helictopleurus nicollei* Lebis, 1960	2008	MZHF	Madagascar
*Helictopleurus perrieri* (Fairmaire, 1898)	2006	MZHF	Madagascar
*Helictopleurus sinuatocornis* (Fairmaire, 1898)	2003	MZHF	Madagascar
*Helictopleurus undatus* (Olivier, 1789)	2008	MZHF	Madagascar
*Helictopleurus* near *furcicornis* Lebis, 1960	2008	MZHF	Madagascar
*Nesosisyphus rotundatus* Vinson, 1946 [holotype]	1944	NHMUK	Mauritius
*Onychothecus tridentigeris* Zelenka, 1992	1985	MNHN	Thailand

### DNA extraction and sequencing

We applied an optimized archival DNA extraction protocol to the 11 historical samples. The protocol described in this peer-reviewed article is published on Protocols.io (DOI 10.17504/protocols.io.81wgbybqyvpk/v3) and is included for printing purposes as (S1 File). Briefly, the protocol is a customization of the archival DNA extraction protocol and Guanidine treatment described by Straube et al. (2021) [22] which was influenced by the studies of Dabney et al. (2013) [30] and Rohland et al. (2004) [31]. The new approach was first proposed for wet-preserved vertebrates and is based on the binding of DNA to a PCR purification silica membrane in the presence of a chaotropic salt (guanidine hydrochloride) buffer (S2 Table and at Protocols.io). The method uses an extension reservoir attached to a commercial silica spin column, able to retain DNA fragments of lengths varying from 70 bp to 4 kbp. This adaptation allows for a more than tenfold increase in the ratio of binding buffer to sample and enhances the recovery of short DNA fragments, typically present in historical samples [22]. We further customized this protocol into a non-destructive extraction using dry-preserved beetle specimens from several museum entomological collections, as specified in step 4 of the protocol. In short, we optimized how samples were prepared for the lysis step by not physically destroying body parts. Extractions from the 11 dry-preserved museum specimens (S1 Table) were undertaken in a dedicated “clean room” for historical samples at MZHF.

The DNA of the 59 wet-preserved museum specimens was extracted using the QIAamp DNA Micro Kit (QIAGEN), following the manufacturer’s protocol. After DNA extraction, dual-indexed paired-end Illumina libraries were prepared and enriched using the UCE Scarabaeinae probe-set Scarab 3Kv1 [29] and sequenced at RAPiD Genomics LLC (Gainesville, FL, U.S.A.) utilizing their high-throughput workflow with proprietary chemistry. Briefly, the DNA was sheared to a mean fragment length of 500 bp, followed by end-repair and A-tailing, incorporation of unique dual-indexed Illumina adaptors, and PCR enrichment. Samples were pooled equimolarly and sequenced on an Illumina NovaSeq 6000 S4 flow cell (2x150 bp).

**Fig 1:**
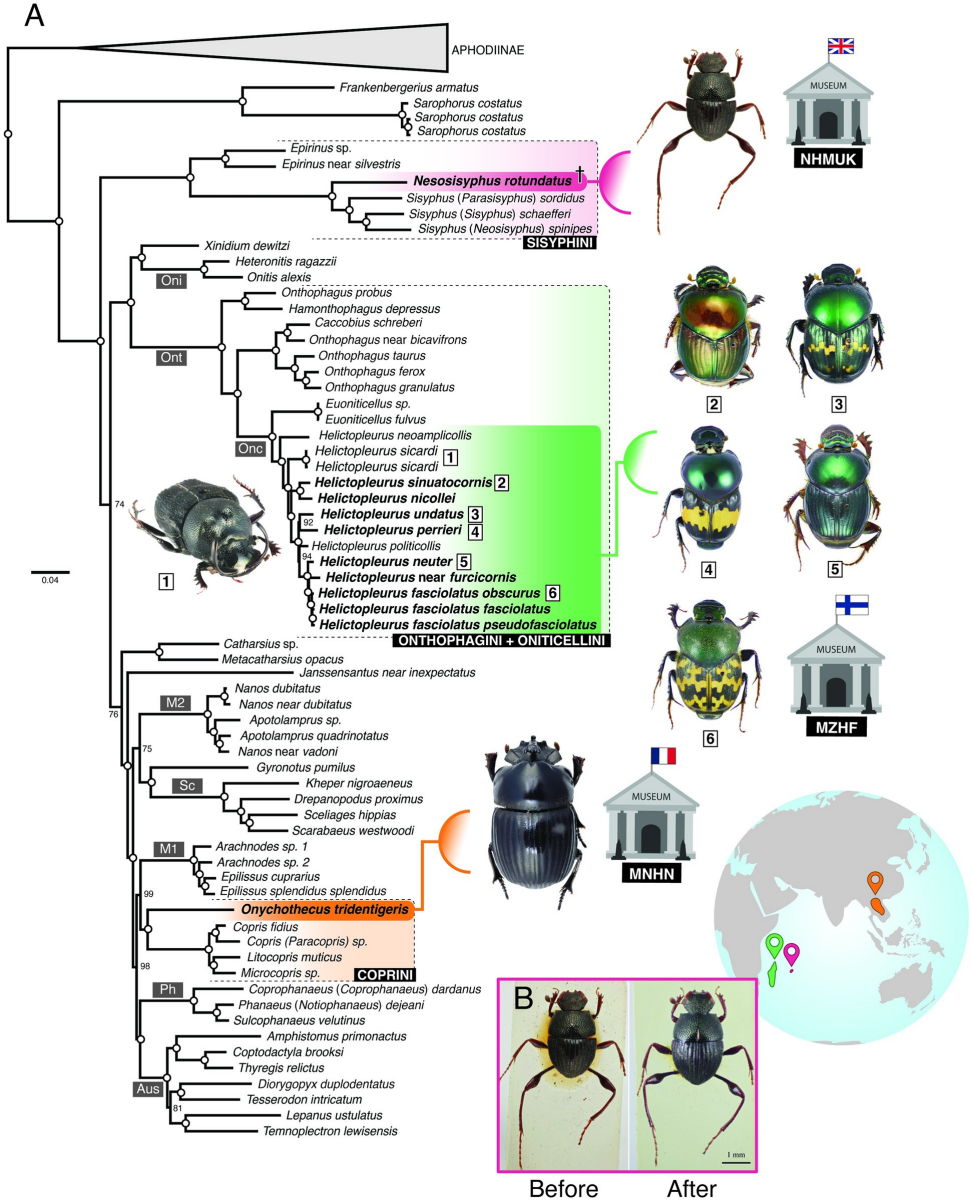
Dung beetle tree. (A) Species tree inferred using 1,497 UCEs and the 50% complete dataset; dry-preserved historical museum specimens are indicated in bold. Collapsed branches are proportional to the number of samples in each lineage. Hollow dots indicate fully supported nodes and nodes with numbers indicate bootstrap values < 100. (Oni) Onitini; (Ont) Onthophagini + Oniticellini; (Onc) Oniticellini; (M2, M1) Madagascan endemic lineages; (Sc) Scarabaeini; (Ph) Phanaeini; (Aus) Australasian endemic genera. For details, see S1 Table. (B) Dorsal view of the holotype of *Neosisyphus rotundatus* before and after DNA extraction using the archival protocol.

**Fig 2:**
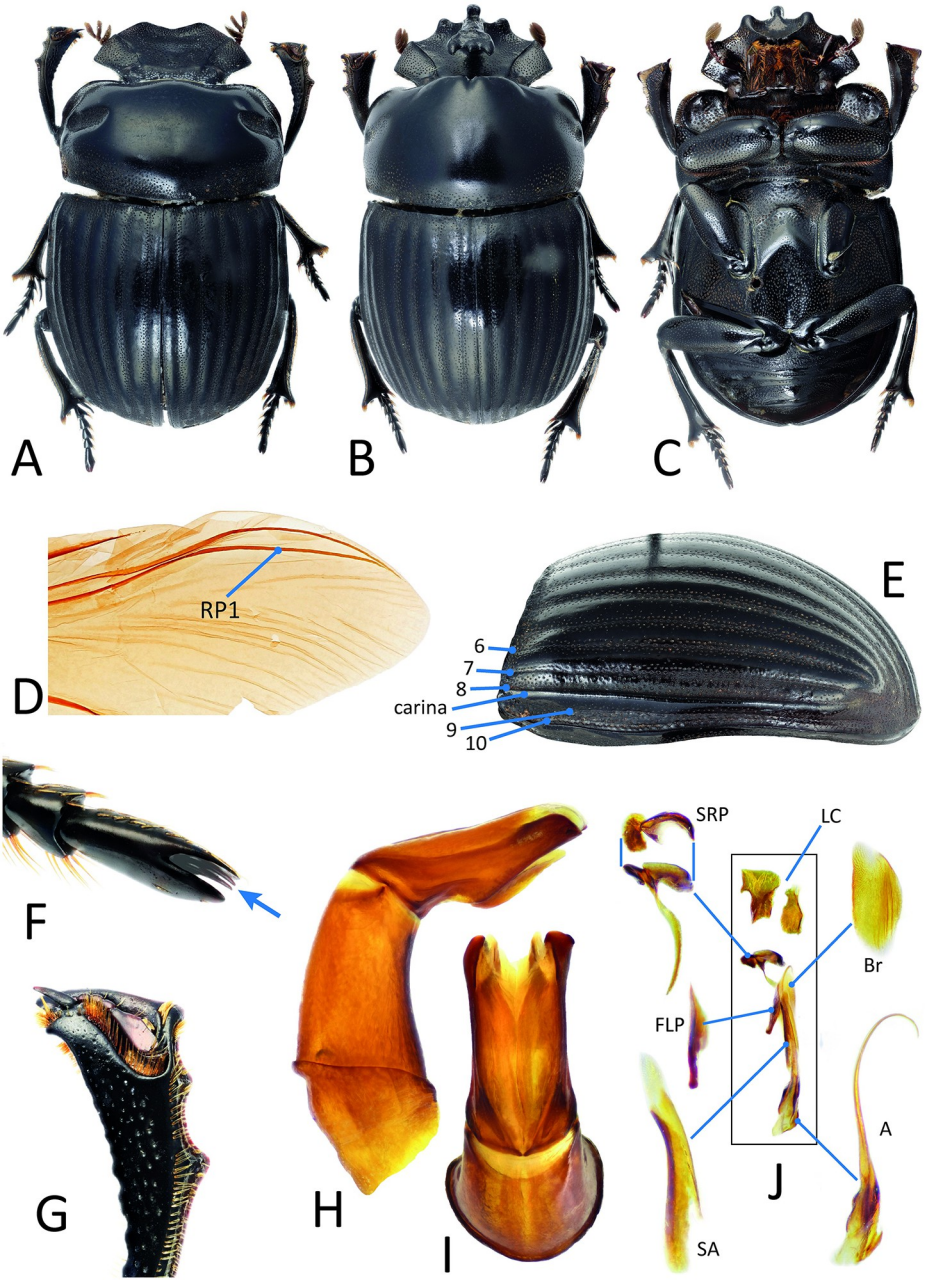
Morphology of *Onychothecus tridentigeris*. Dorsal habitus of male (A) and female (B); ventral view of female (C); right wing in dorsal view, with radial posterior vein 1 (RP1) indicated (D); left elytron in lateral view, indicating the numbered elytral striae and the lateral carina (E); hind tarsus, with the modified terminal tarsomere concealing the claws (F); right protibia of male, in dorsal view (G); aedeagus in lateral (H) and dorsal (I) views; endophallites (J) (abbreviations follow Tarasov & Génier (2015) [33]).

*Nesosysiphus rotundatus*, a monoinsular endemic species from Mauritius that is known only from six pinned specimens, was represented by its holotype, deposited in NMHUK (Fig 1B). The tiny specimen, one of the smallest scarab dung beetles in the world (∼4 mm), was collected by J. Vinson in 1944 [32]. This specimen was carefully relaxed and disarticulated. Only the prothorax (with exposed internal tissues) and the attached forelegs (but not the head) were used during the digestion step of the extraction (Fig 1B), resulting in a total of 17.03 ng of DNA that generated 1,528 UCE loci after sequencing. Following DNA extraction, the digested body parts remained well-preserved with no visible external deterioration, and the specimen was afterward successfully reassembled (Fig 1B). *Onychothecus tridentigeris* Zelenka, 1992 is a much larger, very rare species from Thailand, that was represented by a non-type specimen (∼20 mm) deposited in MNHN (Table 1, Fig 2). We removed the entire left middle leg from the specimen, which was destructively used in the DNA extraction process (Fig 2B and 2C), resulting in 17.40 ng of DNA that generated 1,692 UCE loci. The remainder of the specimen survived intact.

### Data processing

Demultiplexing and trimming were performed by RAPiD Genomics LLC using Illumina bcl2fastq2 2.20 [34] with default settings. Our UCE datasets were assembled using the package Phyluce 1.7.3 [35] following the workflow available on GitHub. Raw demultiplexed reads were first cleaned using Illumiprocessor 2.0 [36] with default parameters set on Phyluce to remove residual adapter contamination. Cleaned reads were inspected for quality using FastQC 0.12.0 [37]. After, cleaned pair-end reads were assembled into contigs with Spades 3.15.4 [38] and default parameters set on Phyluce. The Scarab 3kv1 UCE probes [29] were matched to the assembled contigs in Phyluce, with a minimum identity of 80% and coverage of 80× to avoid off-target contaminating sequences [29, 39]. UCE loci were then extracted from the sequenced data. We harvested UCE loci from the available whole beetle genomes on GenBank [29] (S1 Table. Last access previous data harvesting on 23.05.2023) using using faToTwoBit (https://genome.ucsc.edu/), Phyluce and the Scarab 3Kv1 probe set [29], as described in the Phyluce tutorial, and combined them with our newly sequenced data. The UCE loci were aligned in MAFFT 7.475 [40], using the default Phyluce settings and the command *-no-trim* to provide internal trimming, as recommended for analysis of divergences over 50 million years old [35]. The resulting alignments were parsed to a parallel wrapper around Gblocks 0.91 [41] to eliminate poorly aligned positions and divergent regions using the settings: b1 = 0.5, b2 = 0.85, b3 = 8, b4 = 10 [41, 42]. Summary statistics for the generated datasets were computed using the program AMAS [43].

### Phylogenomics

For phylogenomic analyses, we constructed data matrices for concatenated species trees in IQ-Tree 2.0.7 [44] with 50% and 70% complete data, allowing up to 50% and 30% missing taxa for each locus, respectively [21, 45]. Hereafter, the 50% and 70% complete datasets are named the 50p and 70p datasets. Full and partitioned UCE alignments are available on the Open Science Framework (OSF) repository at DOI 10.17605/osf.io/mxwj7. ID labels in tree files were translated into full species names using the custom Python script *rename_leaves_v1.0.py* available on GitHub.

#### Gene-based phylogeny

Concatenated species trees were estimated in IQ-Tree2 using UCEs as independent loci (genes). Confidence levels were calculated using 1,000 ultrafast bootstrap (UFBoot) replicates and topologies tested by the Shimodaira–Hasegawa test (SH-aLRT) [46, 47]. The best substitution models were automatically selected using ModelFinder (*-m mfp* option) implemented in IQ-Tree2 under the Bayesian Information Criterion [48].

To reduce the risk of overestimating branch support with UFBoot owing to severe model violations, we used a hill-climbing nearest-neighbor interchange (NNI, *-bnni* option) [49] topology search strategy to optimize each bootstrap tree. As phylogenetic models rely on various simplifying assumptions to ease computations (*e.g.*, treelikeness, reversibility and homogeneity of substitution models), estimations of some genomic regions can severely violate model assumptions, causing biases in phylogenetic estimates of tree topologies [50]. To test these violations on each locus, we also applied the test of symmetry with the option --*symtest-remove-bad* [50]. Partitions (concatenated analyses) and genes (species trees analyses) with a *p*-value ≤ 0.05 for the test of symmetry were removed from downstream analyses [50].

#### Partition-based phylogeny

We also recovered concatenated species trees with partitioned genomic regions. The datasets were partitioned with PFinderUCE-Sliding-Window Site Characteristics (SWSC-EN), an entropy-based method developed specifically for UCE data [51]. To implement the SWSC-EN method, we used Phyluce to generate a concatenated *Nexus* file with the location of each UCE locus as character sets. With the SWSC-EN Python 3.6 script, configuration files were created to be processed by Partitionfinder 2.1.1 [52] and Python 2.7. As Partitionfinder2 works only with *Phylip* alignments, we converted the concatenated *Nexus* file to Relaxed *Phylip* format using Geneious 2022.2.1 [53]. The partitioning scheme was then generated with Partitionfinder2 with linked branch lengths, a GTR+G model of evolution, an Akaike information criterion with correction (AICc) [54] for model selection and a variant of the relaxed hierarchical clustering search algorithm https://osf.io/3fpg4/ [21, 55].

### Morphological examination

As a complement to molecular inference of the phylogenetic position of *Onychothecus* and related taxa, we studied the morphology of two available specimens of *O. tridentigeris* (deposited in MNHN) in detail. Morphological terminology and protocols follow Tarasov & Dimitrov (2016) [28] and Tarasov & Génier (2015) [33]. Specimens were examined under a Leica S9D stereomicroscope (Leica Microsystems GmbH, Germany). Photographs were taken with a Canon MP-E 65 mm, f/2.8, 1–5× macro lens mounted on a Canon EOS 5D (Canon Inc., Japan) camera and then stacked using the StackShot (Cognisys Inc., USA) automated system.

## Results

### UCE data

**Fig 3:**
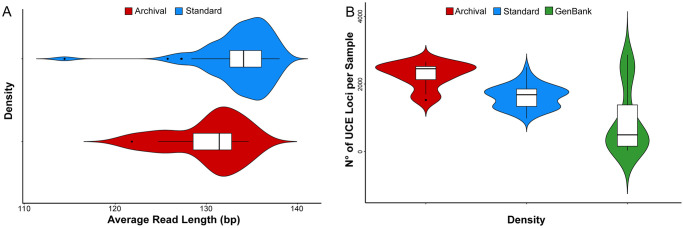
Summary of UCE data resulting from two DNA extraction methods (archival extraction protocol in red and standard extraction in blue) and beetle genomes from GenBank (in green, only in B). Violin plots illustrate the kernel density and boxplots display the median and variation. (A) Distribution of read length generated per sample, demonstrating that the density of shorter reads was generally higher from archival extractions. (B) Distribution of the number of UCE loci per sample, demonstrating that the greatest density of samples that generated large numbers of captured loci resulted from archival extractions.

We obtained a mean of 1.86×10^7^ paired-end reads per sample. Our results revealed that shorter fragments from museum samples were effectively integrated into DNA libraries, resulting in the recovery of a substantial number of UCE loci for phylogenomic analyses (Fig 3A, S1 Table). Specifically, samples for which DNA was extracted using the archival DNA protocol yielded the highest number of recovered loci (2,264), followed by alcohol-preserved samples extracted using the commercial kit (1,620 loci) and UCE data retrieved from GenBank genomes (909 loci; S1 Table and Fig 3B). Interestingly, older specimens, such as *O. tridentigeris* and *N. rotundatus*, yielded a similar number of recovered loci compared to the more recently collected wet-preserved samples extracted with the commercial kit. (Table 1 and S1 Table).

Prior to data filtering, the full concatenated alignment (96 tips) contained 3,160 UCE loci and 269,808 parsimony-informative sites distributed across 675 Kbp (S3 Table). The 50p dataset contained 1,497 UCE loci with a mean of 79.97 parsimony-informative sites per locus (S4 Table) and the 70p dataset contained 289 UCE loci with a mean of 60.54 parsimony-informative sites per locus (S5 Table). The conspicuous disparity among unfiltered, 50p and 70p datasets is due to the large proportion of missing data present in the genomes retrieved from GenBank, which mostly served as outgroup taxa in our study [29] (S2 Table and S1–S5 Figs). UCE data from GenBank-represented species was mostly obtained from existing draft genomes. These genomes, having been assembled in different sizes and showing discrepant levels of contiguity, were the primary contributors to the aforementioned missing data.

### Phylogenomics

Increasing the completeness threshold during construction of data matrices significantly decreased the number of UCE loci and overall bootstrap support (Fig 1, S1–S4 Figs and S4 and S5 Tables). Because phylogenomic studies generally do not benefit from filtering out loci having an increased proportion of missing data [45], we focused on the 50p dataset, which resulted in an optimal trade-off between the highest overall bootstrap support and SH-values (Fig 1 and S1 and S2 Figs) and the number of recovered loci (S1 Table).

Our phylogenetic trees were well-supported, with only a few nodes of moderate depth having poor support (see Fig 1 and S1 and S2 Figs). Notably, Scarabaeinae formed a monophyletic group, with *Frankenbergerius* Balthasar and *Sarophorus* Erichson representing a basal lineage that is sister to the remaining Scarabaeinae. The Afrotropical genus *Epirinus* Dejean was identified as the sister to the remaining Sisyphini. The historical specimen of *N. rotundatus* consistently clustered within the tribe Sisyphini across all analyses, with robust bootstrap support (BS: 100; Fig 1A and 1B).

In all analyses, the Madagascan *Helictopleurus* species formed a sister clade to the other Oniticellini (clade Onc in Fig 1), represented solely by *Euoniticellus* Janssens in our tree. Slight variations in the grouping of certain *Helictopleurus* species resulted from analysis of the 70p dataset, likely due to limited genomic information and/or short branches in the clade’s backbone (Fig 1 and S1–S4 Figs). We recovered the Oniticellini + *Helictopleurus* clade as nested within Onthophagini (clade Ont in Fig 1). The enigmatic species *O. tridentigeris* was consistently inferred in all analyses as the sister taxon to the genera belonging to the tribe Coprini (BS: ≥ 98; Figs 1A and 2).

Other dung beetle tribes were also recovered as monophyletic (Fig 1), namely, Onitini (clade Oni), Scarabaeini (clade Sc), and Phanaeini (clade Ph). The Madagascan “Canthonini” were grouped into two lineages: *Apotolamprus* + *Nanos* (clade M2) and *Arachnodes* + *Epilissus* (clade M1). The lineage M2 was sister to Scarabaeini + *Gyronotus*, while M1 was sister to Coprini. Additionally, the Australasian endemic genera also formed a monophyletic group (clade Aus in Fig 1).

## Discussion

How specimens are captured, killed and preserved prior to being dry-mounted are likely to be crucial factors contributing to DNA quality and subsequent phylogenomic analyses based on these data. In this case, no such information is available for the historical dry-preserved specimens that we extracted, yet it is reasonable to assume a variety of preservation treatments- as is common in any museum collection. For example, the species of *Helictopleurus* were collected in the early 2000s by Ilkka Hanski’s team at the Finnish Museum of Natural History, who probably optimized field sampling protocols to limit DNA damage for anticipated evolutionary studies. Indeed, these *Helictopleurus* specimens resulted in generating a correspondingly large amount of DNA sequence data—an average of 2,410 UCEs per sample (see Results and S1 Table). Conversely, it was expected that the older specimens *Onychothecus* and *Nesosisyphus*, likely collected without molecular-oriented awareness (*e.g.*, by using DNA-damaging killing compounds such as ethyl acetate, widely used by entomologists), would result in significantly diminished DNA data. However, both specimens yielded substantial numbers of UCEs (1,692 and 1,592, respectively) that were sufficient to phylogenetically place them, with strong support, within our scarab beetle tree. This therefore highlights the feasibility of applying our workflow to retrieve and sequence DNA for phylogenomics even from specimens of unknown preservation history.

### Phylogenetic relationships

All resultant topologies were generally consistent with previous morphological [33] and molecular analyses based on individual genes [28, 33, 56] and preliminary UCE data [29].

The basal position of the Afrotropical lineage comprising *Frankenbergerius* + *Sarophorus*, as well as the monophyly of Onitini, Scarabaeini, Phanaeini and Onthophagini + Oniticellini, are consistent with previous findings [28, 33, 56]. Additionally, the paraphyly of Onthophagini with Oniticellini nested within it has also been supported by previous analyses [28, 57–59]. The Old World tribe Onitini (clade Oni in Fig 1) was found to be sister to the Afrotropical genus *Xinidium* Harold, consistent with morphological and mitochondrial data [33, 59], whereas other molecular data tend to place Onitini as sister to Onthophagini + Oniticellini [28, 58] or Sisyphini [56]. Similar discordance between molecular and morphological data was observed in the relationship between Afrotropical *Gyronotus* Lansberge and the Old World tribe Scarabaeini (clade Sc in Fig 1). Molecules suggest a remote relationship [28], while morphology supports close affinities [33]. The splitting of Madagascan “Canthonini” into two lineages, *Apolamprus* + *Nanos* (clade M2) and *Arachnodes* + *Epilissus* (clade M1), has also been supported by previous studies [28, 60]. Additionally, earlier molecular analyses have consistently supported the monophyly of the Australasian endemic genera (clade Aus in Fig 1) included in this study [28, 56]. The relationships of the enigmatic taxa sequenced from historical specimens are discussed in detail below.

**Mauritian *Nesosisyphus*.** This study offers the first insights into relationships of the endemic Mauritian genus *Nesosisyphus*, which has not previously undergone phylogenetic analysis. Our grouping of *N. rotundatus* (Fig 1A) within the tribe Sisyphini [28, 61] was expected and is also supported by morphological synapomorphies that clearly indicate that *Nesosisyphus* is a member of this tribe. Therefore, the phylogenetic result based on UCE data obtained from the old holotype of *N. rotundatus* strongly agrees with our initial hypothesis [32].

*Nesosisyphus rotundatus* is a flightless roller dung beetle, uniquely known by the six specimens that make up the type series collected during the early 1940s from the southern slope of Mount Ory in Mauritius [32]. All subsequent collecting efforts on the island, which have included sampling at the type locality, have resulted in the discovery of three additional Mauritian endemic species of *Nesosisyphus* (Losacco et al., *in prep.*), failed to relocate this species. Given this and considering the rapid loss of indigenous habitats and biodiversity in Mauritius in general, due to anthropogenic habitat destruction and the introduction of exotic species [62, 63], we regard this species as potentially extinct (Losacco et al., *in prep*). One of the achievements of our study has been to unlock genomic data from this enigmatic species for further investigation. An additional benefit of having genomic data available for this species is that further eDNA studies (*e.g.*, metagenomics) could utilize this data to locate the species and determine its distribution using indirect DNA sources, such as soil, through an eDNA approach.

**Oriental *Onychothecus*.** This extremely rare genus comprises four species distributed in southeastern Asia (Figs 1 and 2): China (Yunnan), Myanmar, Thailand, Laos and Vietnam [64, 65]. It is remarkable for displaying secondary sexual dimorphism that is unusual within scarab beetles—females bear a cephalic horn and males are hornless (the reverse is overwhelmingly more common in the superfamily Scarabaeoidea)—in addition to having unknown habits, diet and general biology. *Onychothecus* has not yet been classified (=*incertae sedis*) into any of the existing tribes of the subfamily Scarabaeinae [28]. Only a single previous phylogenetic analysis incorporating this genus exists [66], based upon morphological data that identified it as sister to the genus *Paraphytus*, having a disjunct Afrotropical and Oriental distribution. *Paraphytus* belongs to the most basal lineage of Scarabainae [28] that also includes the Afrotropical genera *Frankenbergerius* and *Sarophorus*, which we have included in the present analyses. Our resulting phylogeny indicates that *Onychothecus* does not belong to that basal lineage, being instead recovered as sister to the clade containing the genera *Copris* Geoffroy, *W*aterhouse and *Microcopris* Balthasar, belonging to the tribe Coprini *sensu* Tarasov & Dimitrov (2016) [28] (Fig 1A and S1–S4 Figs). Consequently, based on our results, we assign *Onychothecus* to the tribe Coprini and discuss this assignment in a separate section below. **Oriental** *Onychothecus*. This extremely rare genus comprises four species distributed in southeastern Asia (Figs 1 and 2): China (Yunnan), Myanmar, Thailand, Laos and Vietnam [64, 65]. It is remarkable for displaying secondary sexual dimorphism that is unusual within scarab beetles—females bear a cephalic horn and males are hornless (the reverse is overwhelmingly more common in the superfamily Scarabaeoidea)—in addition to having unknown habits, diet and general biology. *Onychothecus* has not yet been classified (=*incertae sedis*) into any of the existing tribes of the subfamily Scarabaeinae [28]. Only a single previous phylogenetic analysis incorporating this genus exists [66], based upon morphological data that identified it as sister to the genus *Paraphytus* Harold, having a disjunct Afrotropical and Oriental distribution. *Paraphytus* belongs to the most basal lineage of Scarabainae [28] that also includes the Afrotropical genera *Frankenbergerius* and *Sarophorus*, which we have included in the present analyses. Our resulting phylogeny indicates that *Onychothecus* does not belong to that basal lineage, being instead recovered as sister to the clade containing the genera *Copris* Geoffroy, *Litocopris* Waterhouse and *Microcopris* Balthasar, belonging to the tribe Coprini *sensu* Tarasov & Dimitrov (2016) [28] (Fig 1A and S1–S4 Figs). Consequently, based on our results, we assign *Onychothecus* to the tribe Coprini and discuss this assignment in a separate section below.

**Madagascan *Helictopleurus*.** The genus *Helictopleurus* comprises approximately 65 species, all endemic to Madagascar (Fig 1A) and primarily occurring in forest habitats [60, 67]. We extracted and sequenced DNA from nine dry-preserved and two alcohol-preserved specimens from museum collections, having been collected between 2003 and 2010. Sequence data for one additional species was included from a previously published study [68]. Our phylogenetic analyses (Fig 1A and S1–S4 Figs) confirm previous results, demonstrating the monophyly of the genus *Helictopleurus*, its sister relationship to the genus *Euoniticellus* and that the *Helictopleurus* + *Euoniticellus* clade falls within the clade containing the tribes Onthophagini + Oniticellini [57, 58, 69]. However, when compared to earlier molecular phylogenies based on individual genes, our analyses resulted in slight variations in the interspecific relationships within *Helictopleurus* [68, 69]. Enhancing taxon sampling in future studies and potentially integrating previously published single-gene data will help achieve more robust results.

The fact that our results, including newly sequenced data from museum specimens of varying ages (Table 1) and preservation, produced robust results that are consistent with existing phylogenies [28, 29], demonstrates the effectiveness of the proposed archival DNA approach in combination with UCE sequencing. Such consistency is of particular significance because concerns about sequence data obtained from historical specimens being contaminated or of poor quality and, consequently, obfuscating or impeding phylogenetic inference, appear not to have been borne out in our study.

### Tribal transfer of *Onychothecus* to Coprini

**Genus *Onychothecus* Boucomont, 1912**

- *Onychothecus* Boucomont, 1912: original description; *as member of* Scatonomini Lacordaire, 1856 *synonym of* Deltochilini Lacordaire, 1856: *sensu* Bouchard et al. (2011) [70].
- *Onychothecus*; Balthasar (1963) [71]: *as member of* Pinotini Kolbe, 1905 *synonym of* Ateuchini Perty, 1830: *sensu* Smith (2006) [72].
- *Onychothecus*; Tarasov & Dimitrov (2016) [28]: *as* *incertae sedis*.
- **Type species:** *Onychothecus ateuchoides* Boucomont, 1912.

The tribe Coprini is distributed in both the Old and New World and includes five genera: *Copris*, *Litocopris*, *Microcopris*, *Pseudocopris* Ferreira and *Pseudopedaria* Felsche. The tribe lacks unique apomorphies allowing for unequivocal diagnosis. Instead, it is characterized only by a combination of six characters [28].

We examined the morphology of two specimens of *Onychothecus tridentigeris* in detail, including the first-known male of the species (Fig 2A and 2G–2J). Our phylogenetic analyses strongly support the position of *Onychothecus* as a sister taxon to a clade containing the five genera making up the Coprini. Based upon this evidence, two alternative taxonomic actions were considered: either creating a new tribe to accommodate *Onychothecus* or assigning it to Coprini. We have chosen the latter option and herein treat the genus *Onychothecus* as a member of the tribe Coprini. In our opinion, this action is justified to maintain stability in the classification of Scarabaeinae.

Although the general habitus of *Onychothecus* resembles that of many other members of the tribe Coprini, its morphology stands out within the Scarabaeinae in general. Specifically, *Onychothecus* exhibits dorsally excavated protibial apices, terminal tarsomeres that conceal the tarsal claws and, most strikingly, ‘inverse sexual dimorphism’, wherein the female is the horned sex. Additionally, it possesses characters that have not previously been adopted to diagnose Coprini, including laterally carinate elytra, an absence of the posterior sclerite of the wing, and an absence of the posterior ridge of the hypomera. These observations therefore oblige revision and expansion of the morphological diagnosis of the tribe Coprini. We present the new diagnosis of Coprini in Table 2.

**Table 2:** Updated diagnosis of the tribe Coprini. The combination of characters 1–6 constitutes a diagnosis of Coprini that includes *Onychothecus*; for details see Tarasov & Dimitrov (2016) [28]. Characters 7–10 (marked with *) refer to autapomorphies for *Onychothecus*.

Character	*Onychothecus*	other Coprini genera
1. Wing apex, posterior sclerite	absent (Fig 2D)	present
2. Number of elytral striae	10 (Fig 2E)	10
3. Elytral stria 8	carinate (Fig 2E)	not carinate
4. Superior right peripheral (SRP) endophallite	not ring-shaped (Fig 2J)	not ring-shaped
5. Hypomera, anterior ridge reaches lateral margin	present	present
6. Posterior longitudinal hypomeral ridge	absent	usually present
7. Last tarsomere	concealing tarsal claws (Fig 2F–2G)	not concealing tarsal claws
8. Protibial apex	excavated dorsally (Fig 2G)	not excavated dorsally
9. Parameres	asymmetric (Fig 2H–2I)	usually symmetric
10. Sexual dimorphism	only females with cephalic horn	often males with cephalic horn

## Conclusion

We successfully obtained genomic data that allowed for the evolutionary positioning of several dung beetle species represented by unique historical specimens deposited in important natural history museum collections (Table 1). Our results were consistent with previous phylogenetic studies [28, 29, 33, 56] and demonstrate that combining a minimally destructive and low-cost archival DNA extraction, with subsequent target enrichment of DNA libraries for sequencing a curated set of beetle UCE loci, is an efficient museomics tool for phylogenomics (see S1 and S2 Tables). The proposed extraction protocol should also combine well with Anchored Hybrid Enrichment sequencing. By being able to capture small fragments of degraded DNA even from the limited quantity of source tissue available in old museum specimens (Fig 3), we have demonstrated a favorable trade-off between preserving specimen morphology and generating informative genomic-level data. Our customized extraction protocol can be performed using standard equipment commonly available in molecular laboratories within two days, including an overnight digestion step. Because the procedure is designed to capture small amounts of fragmented DNA, prone to cross-contamination, simultaneous handling of a large number of samples is not recommended and the protocol is therefore optimized for 4–6 samples in each extraction batch (see protocol) to minimize this risk. We believe that the method’s strength is that it is particularly applicable when extractions from old specimens deposited in museum collections are necessary. Because many taxa are rare and known only by unique or very few valuable specimens held in museums, non-destructive museomics methods, such as the one we have described, are essential to allow for such (often inordinately interesting) taxa to be included in phylogenomic studies.

## Supporting information

S1 File Step-by-step protocol, also available on protocols.io. This protocol follows the guanidine treatment protocol by Straube et al. (2021) [22], based on Dabney et al. (2013) [30] and Rohland et al. (2004) [31].
(PDF)

S1 Table Sample and sequencing summary information. Genus and species information along with associated taxonomic details, repository, lab codes, and genetic data.
(XLSX)

S2 Table Consumables and permanent material. Detailed information on plastics, chemicals and permanent material used in this study to perform the archival DNA extraction protocol.
(XLSX)

S3 Table Summary statistics of genetic data. Alignment-specific metrics, detailing various statistical measures for the UCE loci concatenated alignment.
(XLSX)

S4 Table Summary statistics of genetic data. Alignment-specific metrics, detailing various statistical measures for UCE loci alignments from 50p dataset.
(XLSX)

S5 Table Summary statistics of genetic data. Alignment-specific metrics, detailing various statistical measures for UCE loci alignments from 70p dataset.
(XLSX)

S1 Fig Gene-based phylogeny. Phylogeny reconstructed with 50p dataset containing all taxa assessed in this study and bootstrap/SH values.
(TIF)

S2 Fig Partition-based phylogeny. Phylogeny reconstructed with 50p dataset containing all taxa assessed in this study and bootstrap/SH values.
(TIF)

S3 Fig Gene-based phylogeny. Phylogeny reconstructed with 70p dataset containing all taxa assessed in this study and bootstrap/SH values.
(TIF)

S4 Fig Partition-based phylogeny. Phylogeny reconstructed with 70p dataset containing all taxa assessed in this study and bootstrap/SH values.
(TIF)

S5 Fig Matrix showing the presence of loci (black) for each sample. The absence is shown in white. The amount of missing data is highlighted with red arrows and rectangles for UCEs captured from genomes from Genbank.
(TIF)
