# Supplementary material for: From museum drawer to tree: Historical DNA phylogenomics clarifies the systematics of rare dung beetles (Coleoptera: Scarabaeinae) from museum collections
Source: PLoS One. 2024 Dec 31;19(12):e0309596. doi: 10.1371/journal.pone.0309596 (PMC11687894; doi:10.1371/journal.pone.0309596)
Supplement: S1 File — This protocol follows the guanidine treatment protocol by Straube et al. (2021) [22], based on Dabney et al. (2013) [30] and Rohland et al. (2004) [31]. (PDF) [file pone.0309596.s001.pdf]

Aug 20, 2024 Version 3

Archival DNA extraction protocol for insect specimens from museum collections V.3

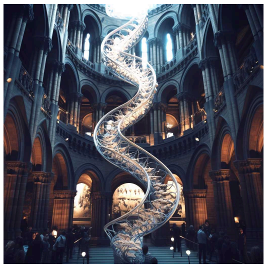

DOI
dx.doi.org/10.17504/protocols.io.81wgbybqyvpk/v3

Fernando Lopes[1], Nicole Gunter[2], Conrad P. D. T. Gillett[1], Giulio Montanaro[1], Michele Rossini[3], Federica Losacco[1], Gimo M. Daniel[4], Nicolas Straube[5], Sergei Tarasov[1]

[1]Finnish Museum of Natural History - University of Helsinki; [2]Queensland Museum Kurilpa, Brisbane; [3]University of Padova; [4]National Museum, Bloemfontein; [5]University Museum of Bergen

Tarasov' Lab

Fernando Lopes
University of Helsinki

OPEN ACCESS

DOI: dx.doi.org/10.17504/protocols.io.81wgbybqyvpk/v3

Protocol Citation: Fernando Lopes, Nicole Gunter, Conrad P. D. T. Gillett, Giulio Montanaro, Michele Rossini, Federica Losacco, Gimo M. Daniel, Nicolas Straube, Sergei Tarasov 2024. Archival DNA extraction protocol for insect specimens from museum collections. **protocols.io https://dx.doi.org/10.17504/protocols.io.81wgbybqyvpk/v3**Version created by **Fernando Lopes**

License: This is an open access protocol distributed under the terms of the **Creative Commons Attribution License**, which permits unrestricted use, distribution, and reproduction in any medium, provided the original author and source are credited

Protocol status: Working
We use this protocol and it's working

Created: April 24, 2023

Last Modified: August 20, 2024

Protocol Integer ID: 104782

Keywords: Lysis, 2-mercaptoethanol, Phylogenomics, Filtration

Funders Acknowledgement:
Nicole Gunter
Grant ID: DEB-1942193
Sergei Tarasov
Grant ID: #331631
Sergei Tarasov
Grant ID: #79783104
Conrad P. D. T. Gillett
Grant ID: Pentti Tuomikoski
Fund 2023

Abstract

This protocol can be used to dry specimens from natural history collections to obtain DNA for UCE-seq.
The protocol follows the guanidine treatment protocol by Straube et al. (2021), if applying this protocol cite and read the corresponding references from the References section.

Attachments

PDF
men13433-sup-0001-ap...
1.2MB

PDF
Molecular Ecology Re...
664KB

Guidelines

Fundamental reading before starting

From museum drawers to a tree: phylogenomics of historical DNA sheds light on the systematics of rare dung beetles (Coleoptera: Scarabaeinae) from museum collections

Authors: Fernando Lopes, Nicole Gunter, Giulio Montanaro, Michele Rossini, Federica Losacco, Conrad P.D.T. Gillett, Gimo Daniel, Nicolas Straube and Sergei Tarasov

This protocol follows the guanidine treatment protocol by Straube et al. (2021), based on Dabney et al. 2013 and Rohland et al. 2004.

If applying this protocol cite and read the references mentioned above and in the manuscript.

We have successfully extracted and sequenced Ultra Conserved Elements (UCEs) from specimens deposited in museum collections for more than 40 years.

The adapted part of this protocol is described in steps 4 and 5.

More tips below:

- **Tween-20:** Stock concentration (100%) is impossible to pipette. Make a 10% solution before starting using a small becker. 20-50 ml is a good amount to keep in the lab if you use it with some frequency. Avoid the light, since the 100% tween-20 comes inside an amber bottle.
- **Non-destructive buffer**: 1 sample = 1 ml of non-destructive buffer
- Always add one extra sample to the calculation to give you flexibility while setting up your buffer!
- 2-mercaptoethanol must be added just prior to use! in a fume hood. Gently invert it 3 times to mix.
- Set the oven to 37 ºC. Your rotator must fit inside the oven. Our DNA lab has this brand: Boekel Scientific Mini Tube Rotator, 260750

Materials

List of Lab Equipment & Reagents:

Checklist of consumables:

A	B	C
**Products used**	**Company**	**Catalogue number**
10-200 ul filter tips	Sartorius	Z757764-960EA; Z757799-960EA
100-5,000 ul tips	Sartorius	780304; 780300
Axygen Tube 2.0 ml	Axygen	12659585
Axygen Tube 1.5 ml	Axygen	11351904
Zymo column	BioSite	C1016-50
Weighing Boats	Fisherbrand	11344125
Single use scalpels	Any	
1.5 ml Low Retention Tube	ThermoScientific	11569914
Falcon Tube 15 and 50 ml	Fisher	E1450-0200; E1415-0200
Nitril Gloves	Ansell TouchNTuff	11726584
LLG-Spoon spatulas, 18	Labnet	9150800
Ethanol 70-96%	Any	
Bleach 10% for cleaning bench and equipment	Any	
DNA Away surface decontaminant	Thermo Scientific	10223471

filter pipette tips **Sartorius Catalog #**Z757799-960EA

Racked Pipette Tips **Sartorius Catalog #**780304

Axygen™ MaxyClear Snaplock Microtubes, 2.0 mL **Fisher Scientific Catalog #**12659585

Axygen™ MaxyClear Snaplock Microtubes, 1.5 mL **Fisher Scientific Catalog #**11351904

Zymo-Spin Columns **Zymo Research Catalog #**C1016-50

Polystyrene Square-Shaped Standard Weighing Boat **Fisher Scientific Catalog #**11344125

Snap Cap Low Retention Microcentrifuge Tubes **Thermo Fisher Scientific Catalog #**11569914

DNA AWAY™ Surface Decontaminant **Thermo Scientific Catalog #**10223471

Checklist of reagents:

A	B	C
**Reagent used**	**Company**	**Catalog number**
Guanidine thiocyanate (GuSCN)	FisherScientific	10503345
NaCl 5M	FisherScientific	10609823
Tris-HCL 1M pH 8.0	Invitrogen	15568-025
EDTA 0.5M pH 8.0	FisherScientific	10135423

A	B	C
Tween-20 100%	Bio RAD	1706531
HPLC water	Fisher	10367171
Guanidine hydrochloride	Fisher	10543325
Isopropanol	Any	
Sodium Acetate 3M	Fisher	J61928
MiniElute Kit (containing PE buffer)	Qiagen	28006

Guanidinium thiocyanate **Fisher Scientific Catalog #**10503345

NaCl (5 M), RNase-free **Thermo Fisher Scientific Catalog #**10609823

UltraPure™ 1M Tris-HCI pH 8.0 **Thermo Fisher Scientific Catalog #**15568025

EDTA (0.5 M), pH 8.0, RNase-free Invitrogen Molecular biology g **Invitrogen - Thermo Fisher Catalog #**10135423

Tween 20 100% Nonionic Detergent **Bio-Rad Laboratories Catalog #**1706531

Water, HPLC for Gradient Analysis **Fisher Scientific Catalog #**10367171

Guanidine hydrochloride **Fisher Scientific Catalog #**10543325

Sodium acetate, 3M **Fisher Scientific Catalog #**J61928

MinElute PCR Purification Kit **Qiagen Catalog #**28006

TET Buffer:

A	B	C
**Reagents**	**Final concentration**	**Amount for 50ml**
Tris-HCL 1 M ph 8.0	10 mM	500 ul
EDTA 0.5 M pH 8.0	1 mM	100 ul
10% Tween-20	0.05 %	250 ul
ddH2O	-	up to 50 ml

Biding buffer:

A	B	C
**Reagents**	**Final concentration**	**Amount for 50 ml**
Guanidine hydrochloride (MW 95.53)	5 M	23.9 g
Isopropanol	40%	20 ml
10% Tween-20	0.05%	250 ul
Sodium Acetate 3 M	90 mM	1.5 ml
ddH2O	-	up to 50 ml

Non-destructive buffer:

A	B	C
**Reagents**	**Final concentration**	**Amount for 1 sample (1 ml)**
GuSCN powder pure	5 M	0.59 g
NaCl 5M	25 mM	5 ul
Tris 1M ph 8.0	50 mM	50 ul
EDTA 0.5 M pH 8.0	20 mM	44.4 ul
10% Tween-20	1%	100 ul
ddH2O	-	up to 1 ml
2-mercaptoethanol	1%	10 ul (in the fume hood)

Safety warnings

- Use Nitrile gloves in the steps you need to directly touch 2-mercaptoethanol. This chemical is harmful! Do not forget to conduct steps that require 2-mercaptoethanol in a fume hood.
- Change your gloves as quickly as possible and how many times you judge necessary.

Before start

- The protocol can be performed in two days.
- On the first day, you prepare your samples for overnight lysis and prepare plastic ware for overnight UV bath.
- The following day is used for filtering, washing, and elution steps.
- Buffers must be prepared beforehand (check item 3 carefully). Some buffers can be stored for long times, others are just for one-time use.

Preparation for laboratory work

25m

1

Note

The protocol should be conducted in a dedicated room for archival DNA. See Fulton & Shapiro (2019) for a best-practice example.

Clean the bench and all instruments with DNA contamination removal solution and UV light before starting the experiment.

2 **Preparation of buffers:**

2.1 **TET Buffer:**
Can be kept indefinitely

Note

TIPS: Suggested volume 50 mL
UV irradiate before use

A	B	C
**Reagents**	**Final concentration**	**Amount for 50ml**
Tris-HCL 1 M ph 8.0	10 mM	500 ul
EDTA 0.5 M pH 8.0	1 mM	100 ul
10% Tween-20	0.05 %	250 ul
ddH2O	-	up to 50 ml

2.2 **Binding buffer:**
Can be stored for up to 1 month

5m

Note

TIPS: Guanidine may be difficult to dissolve.

- It will take 00:05:00 or more to dissolve all the guanidine. Do it by inverting gently manually in a Falcon tube or small bottle.
- Try to avoid lumps of guanidine.
- Think about the amount of binding buffer you will use in an interval of 1 month as it takes some time to set up the buffer.

A	B	C
**Reagents**	**Final concentration**	**Amount for 50 ml**
Guanidine hydrochloride (MW 95.53)	5 M	23.9 g
Isopropanol	40%	20 ml
10% Tween-20	0.05%	250 ul
Sodium Acetate 3 M	90 mM	1.5 ml
ddH2O	-	up to 50 ml

2.3 **Preparation of buffers for the guanidine protocol:**

Note

IMPORTANT NOTE: The extraction buffer contains 2-mercaptoethanol. go to step #4 must be performed in a fume hood and corresponding safety measures should be met. The 2-mercaptoethanol and Guanidine thiocyanate buffer waste should be discarded appropriately.

Non-destructive buffer

1. **One-time use buffer**

- Guanidinium thiocyanate buffer is adapted from Rohland et al. (2004) but DTT is replaced with 2-mercaptoethanol and Triton X100 with Tween-20.
- Prepare a fresh buffer each time.

Note

TIPS:

- 1 sample = 1 mL of non-destructive buffer
- Always add one extra sample to the calculation to give you flexibility while setting up your buffer! Also, remember to add 2 samples as the negative controls. One is to be placed at the beginning of the row and the other is to be added at the end of the row. **Negative controls and the additional sample will totalize 3 extra samples. A positive control may also be added.**
- 2-mercaptoethanol must be added just prior to use! Gently invert it 3 times to mix.
- We recommend starting with a limited number of samples until you get experienced.

A	B	C
**Reagents**	**Final concentration**	**Amount for 1 sample (1 ml)**
GuSCN powder pure	5 M	0.59 g
NaCl 5M	25 mM	5 ul
Tris 1M ph 8.0	50 mM	50 ul
EDTA 0.5 M pH 8.0	20 mM	44.4 ul
10% Tween-20	1%	100 ul

A	B	C
ddH2O	-	up to 1 ml
2-mercaptoethanol	1%	10 ul (in the fume hood)

2.4 **Binding apparatus** 20m

- Zymo V column extension reservoir (without column)
- Qiagen MinElute silica spin column
- 50 ml Falcom tube
- Pen marker

1. Remove Zymo spin column from the reservoir and submerge the reservoir in bleach for over 00:20:00 . Rinse it well with ddH_2O. Dry it out naturally, preferably in a fume hood. Zymo spin column can be discarded.
2. Cut off the caps of Qiagen MinElute columns and keep the collection tube and caps for the next steps. The caps can be placed on the top of the collection tube to help to maintain sterility.
3. Assemble the binding apparatus by forcibly attaching the Qiagen MinElute (without caps) column in the place reserved for the Zymo spin column. This step can require some force. Do not pressure the region close to the silica membrane.
4. Label the side and top of the Falcon tube to ensure that samples will not be mixed up.
5. UV irradiate it Overnight with the equipment described in go to step #8 .

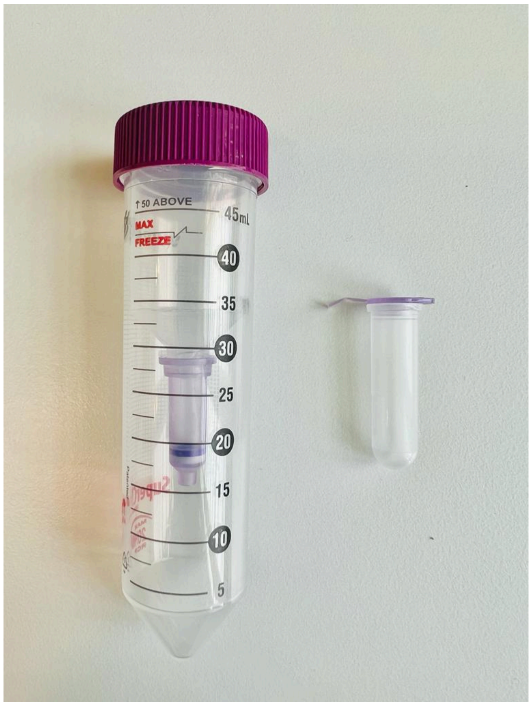

Note

Note: Zymo extended reservoir has been replaced in Rohland et al. 2018 [(Preassembled silica spin columns and collection tubes (High Pure Viral Nucleic Acid Large Volume Kit; Roche, cat. no. 5114403001)]. This information was included under the peer reviewer's request and was not tested by the manuscript's authors.

DNA extraction (protocol's adaptation)

30m

3

Note

The following steps will guide you through the lysis of tissues. This part is adapted from the mentioned references for dry specimens of museum collections.

4 **UV irradiate for** 00:30:00 **:** 30m

- Pieces of parafilm to wrap up caps of 2 ml tubes
- Scissors
- A rack for 1.5 - 2 ml tubes
- 1 15 ml Falcon tube for non-destructive buffer
- Tip Boxes 10, 100/200, and 1,000 ul
- Pipettes
- Pen marker
- Rotator's carousel

5 Clean the bench with bleach or DNA contamination removal solution.

6 For the lysis, you can proceed as below described.

- We have extracted suitable amounts of DNA from different sources.
- However, the yield can vary and sometimes the concentration of DNA can be lower than required for library preparation protocols.

6.1 **Leg or legs of specimens:**

- Depending on the size and structure of the leg(s) and the amount of muscles exposed you can keep the legs intact.
- In this case, the efficiency (amount of DNA retrieved) might be lower; or you can macerate the leg(s) or part of the legs with a sterile lab stick.
- More than one leg of the same individual can be used to increase the total amount of DNA extracted.

Note

- After the extraction, clean well the body parts with ddH_2O. Remember that body parts will stay Overnight in buffer containing 2-mercaptoethanol, a harmful chemical.
- Then remember to rinse the parts in a fume hood with a sink by also wearing nitrile gloves.
- Discard gloves after touching the reagent. With the support of a drying tissue paper and/or naturally dry out the parts. Parts then will be ready to be pinned back.

6.2 **Body parts:**

- With the support of sterile/disposable scalpels, pipette tips or pins you can also use detached body parts. You can leave body parts on lysis in the non-destructive buffer Overnight .
- For dung beetles, we have used different body parts (prothorax, head, abdomen, and legs), that fit 2 ml tubes, with success without causing any damage to external structures, even microstructures.
- Keep in mind that some body parts can contain more sensible parts like antennae.
- Try to avoid body parts that can get stuck in the tube. This can result in lower efficiency or can damage body parts when removing them from the tubes.

Note

- After the extraction, clean well the body parts with ddH_2O. Remember that body parts will stay Overnight in buffer containing 2-mercaptoethanol, a harmful chemical.
- Then remember to rinse the parts in a fume hood with a sink by also using nitrile gloves.
- Discard gloves after touching the reagent. With the support of a drying tissue paper and/or naturally dry out the parts. Parts then will be ready to be pinned back.

6.3 **Internal content:**

- Depending on the size of your specimen you can remove internal content for DNA extraction. Again, with the support of sterile/disposable scalpels, pipette tips or pins you can detach your specimen and extract internal tissues.
- Keep in mind that in this case you will be also extracting DNA from the microbiota, which can bias the DNA concentration estimations and sequencing.

Note

TIPS: We do recommend trials and adaptations based on the type of material you will be extracting DNA from. Use different sizes of body parts to have a general idea of how much DNA you can extract from your specimens.

Note

Label 2 ml tubes accordingly in the caps and walls.

7 **The** Overnight **lysis:**

- Now that you have selected the best tissues for your batch of extractions and before submerging your material in the non-destructive buffer, rinse them with ethanol (except for the internal content) and put them into a 2 ml tube. Leave the caps open and cover the tubes with drying tissue paper to dry out the parts naturally. Tubes containing internal content can be kept closed.
- While your samples are drying, prepare the non-destructive buffer in a Falcon tube of 15 ml as described in go to step #2.3 for your amount of samples + 2 negative controls and the extra sample.

Note

DO NOT ADD 2-mercaptoethanol now. The step before adding 2-mercaptoethanol might be done on a regular bench.

Note

NOTE: This buffer must be made each time and the others should be prepared beforehand.

- Move the material described in step 4 and the non-destructive buffer to the fume hood.
- Put on nitrile gloves and close the lid of the fume hood in a way you can still work, but as closed as possible.
- Carefully add up the 2-mercaptoethanol (10 µL per sample) to the 15 ml Falcon tube containing the non-destructive buffer. You will see a (soft) chemical reaction when both liquids get in touch. Close the Falcon tube and the bottle containing the 2-mercaptoethanol immediately. Invert the tube gently 3 times to mix both buffer and reagent.
- Open the 2 ml tubes containing the negative controls and samples and add 1 mL of non-destructive buffer containing 2-mercaptoethanol to each tube. Close the tubes and seal them with parafilm to avoid leakage.
- Set the tubes evenly in the rotator carousel and place the rotor inside the oven at 37 °C for the Overnight step. The rotator must turn around gently. The cable is ok pinched in the door in most cases.
- Change your gloves.

8 **UV irradiate** Overnight **:**

Note

NOTE: LABEL ALL TUBES (LID AND WALLS) BEFORE TURNING THE UV LIGHT ON.

- 15 ml tubes with 13 mL binding buffer
- MinElute column tubes
- Binding apparatus in 50 ml Falcon tubes (see above)
- 1.5 ml low retention tubes for final extracts
- An aliquot of TET buffer (suggestion 1.5 mL in a 2 ml tube)
- Extra MinElute collection tubes with caps on the top

You can also UV irradiate other equipment. It is up to you!

- Pipettes
- Racks

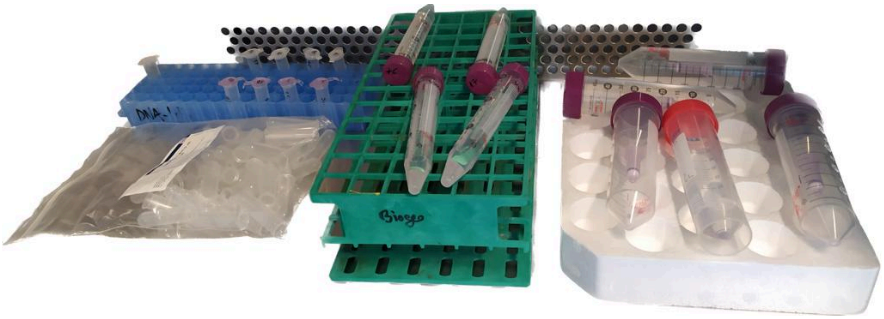

Example of a set up for overnight UV.

Filtering and washing

16m 30s

9 **The next day: after Overnight lysis and UV on plastic ware:**

9.1

Note

Put on nitrile gloves and work in the fume hood in the following steps.

Change nitrile gloves as much as needed. For the samples macerated with sterile sticks or internal tissues removed from specimens, centrifuge for two minutes at maximum speed (~ 15600 g) to pellet tissues. For entire detached parts, skip to the next go to step #9.2 .

9.2 Carefully, add the supernatant to the respective labeled Falcon tube containing the binding buffer, close the lid, and mix by inverting the tube 3 times.

- Pour the buffer with the pellet into the apparatus reservoir. Tape the lids onto the binding apparatus.

> **Note**
>
> Repeat the procedure for all the samples.

9.3 Centrifuge the binding apparatus for 940 x g, 00:04:00 (1,500 rpm). Remember to tape the lids on. Rotate 90º and centrifuge again at the same speed and time. 4m

- You can repeat the procedure a couple of times if there is still liquid in the reservoir (the part above or in the column). You can carefully increase the speed, however, there is the risk that the MinElute column detaches from the reservoir.

> **Note**
>
> **Worse:** you can break the 50 ml tube and spoil harmful liquid inside the centrifuge.

- If after 3 or 4 times of centrifugation you still find some liquid inside you may discard the liquid in the proper waste and proceed with the protocol.

9.4 Prepare 3 new rows of collection tubes (2 ml collection tubes) in the same rack with the collection tubes+lids from the previous day for the next steps.

9.5 Take the filtered material to the fume hood. Detach the MinElute column from the binding apparatus, put them in the original 2 ml collection tubes, and cover them with the lids (already labeled). 1m

- You must have retained them from earlier. Discard the waste properly.
- Dry spin for 3300 rpm, 00:01:00 (6,000 rpm).
- For some samples, a higher speed can be necessary if the liquid does not pass through the column.

9.6 Change the columns to a new row of collection tubes. Discard the flow through properly.

> **Note**
>
> Change your gloves!

9.7 From now on you can work on the regular bench. Wash the silica membrane by adding 650 µL of PE buffer that came with MinElute columns. Centrifuge the tubes at 900 x g (3,300 rpm).

9.8 Repeat step 9.7 once.

9.9 Dry spin columns at maximum speed (~ 15600 x g) for 00:01:00 . Place the columns in clean and labeled 1.5 ml low-retention tubes. 1m

9.10 Elute by adding 12.5 µL TET buffer to the center of the silica membrane. 10m 30s

- Incubate for 00:10:00 , and centrifuge at maximum speed for 00:00:30 .
- Repeat to give a total of 25 µL DNA extract. You must incubate for 00:10:00 in the repetition too.

Concentration and purity measurements

10 We advise the use of Qubit High-Sensitive reagents and 1 µL - 2 µL of extract for DNA concentration measurements. In addition, Nanodrop can be used for purity rate estimations.

Protocol references

1. Straube, N., Lyra, M. L., Paijmans, J. L., Preick, M., Basler, N., Penner, J., ... & Hofreiter, M. (2021). Successful application of ancient DNA extraction and library construction protocols to museum wet collection specimens. *Molecular Ecology Resources*, *21*(7), 2299-2315.

2. Basler, N., Xenikoudakis, G., Westbury, M. V., Song, L., Sheng, G., & Barlow, A. (2017). Reduction of the contaminant fraction of DNA obtained from an ancient giant panda bone. BMC Research Notes, 10(1), 754. https://doi.org/10.1186/s13104-017-3061-3.

3. Dabney, J., Knapp, M., Glock, I., Gansauge, M., Weihmann, A., Nickel, B., Valdioserad, C., García, N., Pääbo, S., Arsuag, J. & Meyer, M. (2013). Complete mitochondrial genome sequence of a Middle Pleistocene cave bear reconstructed from ultrashort DNA fragments. PNAS, 110(39), 15758-15763.

4. Rohland, N., Siedel, H., & Hofreiter, M. (2004). Nondestructive DNA extraction method for mitochondrial DNA analyses of museum specimens. Biotechniques, 36(5), 814-821.
Rohland, N., Hofreiter, M. (2007). Comparison and optimization of ancient DNA extraction. Biotechniques, 42(3), 343–352.

5. Rohland, N., Hofreiter, M. (2007). Ancient DNA extraction from bones and teeth. Nature Protocols, 2, 1756–1762. https://doi.org/10.1038/nprot.2007.247

6. Rohland, N., Siedel, H., & Hofreiter, M. (2010). A rapid column-based ancient DNA extraction method for increased sample throughput. Molecular Ecology Resources, 10, 677–683.

7. Rohland, N., Glocke, I., Aximu-Petri, A., & Meyer, M. (2018). Extraction of highly degraded DNA from ancient bones, teeth and sediments for high-throughput sequencing. Nature Protocols, 13(11), 2447-2461. doi: 10.1038/s41596-018-0050-5.

8. Fulton, T. L., & Shapiro, B. (2019). Setting Up an Ancient DNA Laboratory. In B. Shapiro, A. Barlow, P. Heintzman, M. Hofreiter, J. Paijmans, & A. Soares (Eds.), Ancient DNA: Methods in Molecular Biology (pp. 1–13). Humana Press. https://doi.org/10.1007/978-1-4939-9176-1_1
