# Supplementary figures and images for: From museum drawer to tree: Historical DNA phylogenomics clarifies the systematics of rare dung beetles (Coleoptera: Scarabaeinae) from museum collections
Source: PLoS One. 2024 Dec 31;19(12):e0309596. doi: 10.1371/journal.pone.0309596 (PMC11687894; doi:10.1371/journal.pone.0309596)

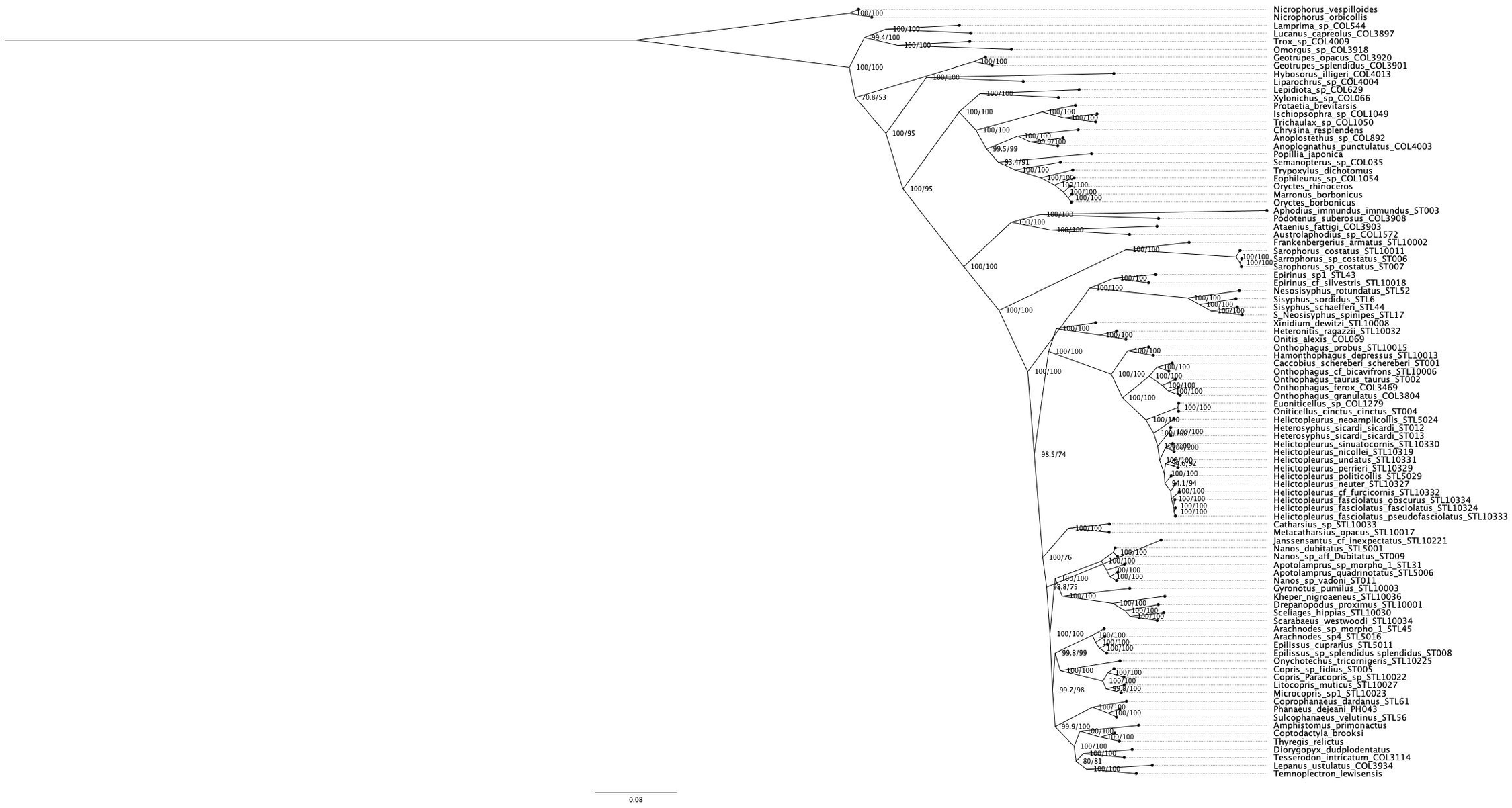

Supplement: S1 Fig — Phylogeny reconstructed with 50p dataset containing all taxa assessed in this study and bootstrap/SH values. (TIF) [file pone.0309596.s007.tif]

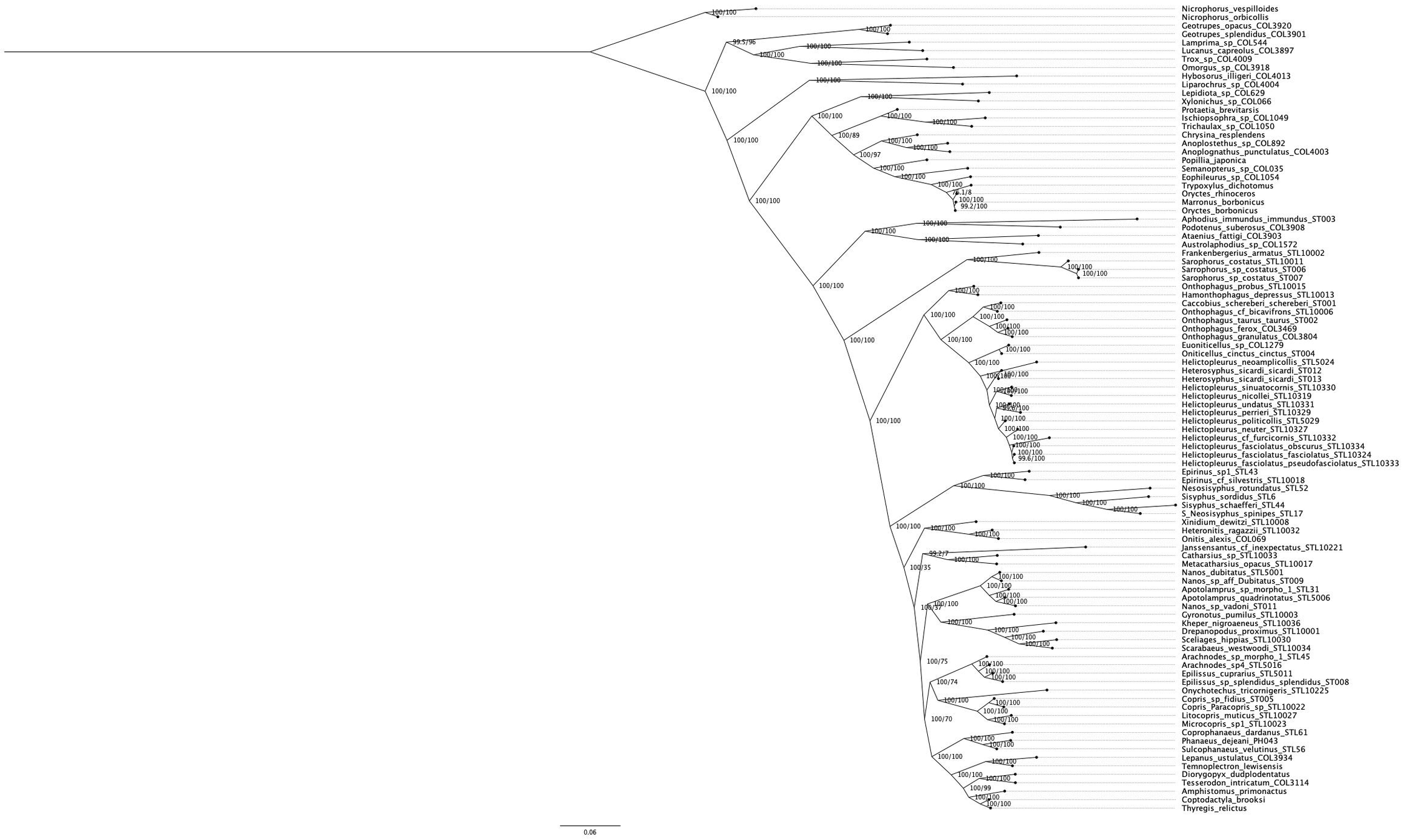

Supplement: S2 Fig — Phylogeny reconstructed with 50p dataset containing all taxa assessed in this study and bootstrap/SH values. (TIF) [file pone.0309596.s008.tif]

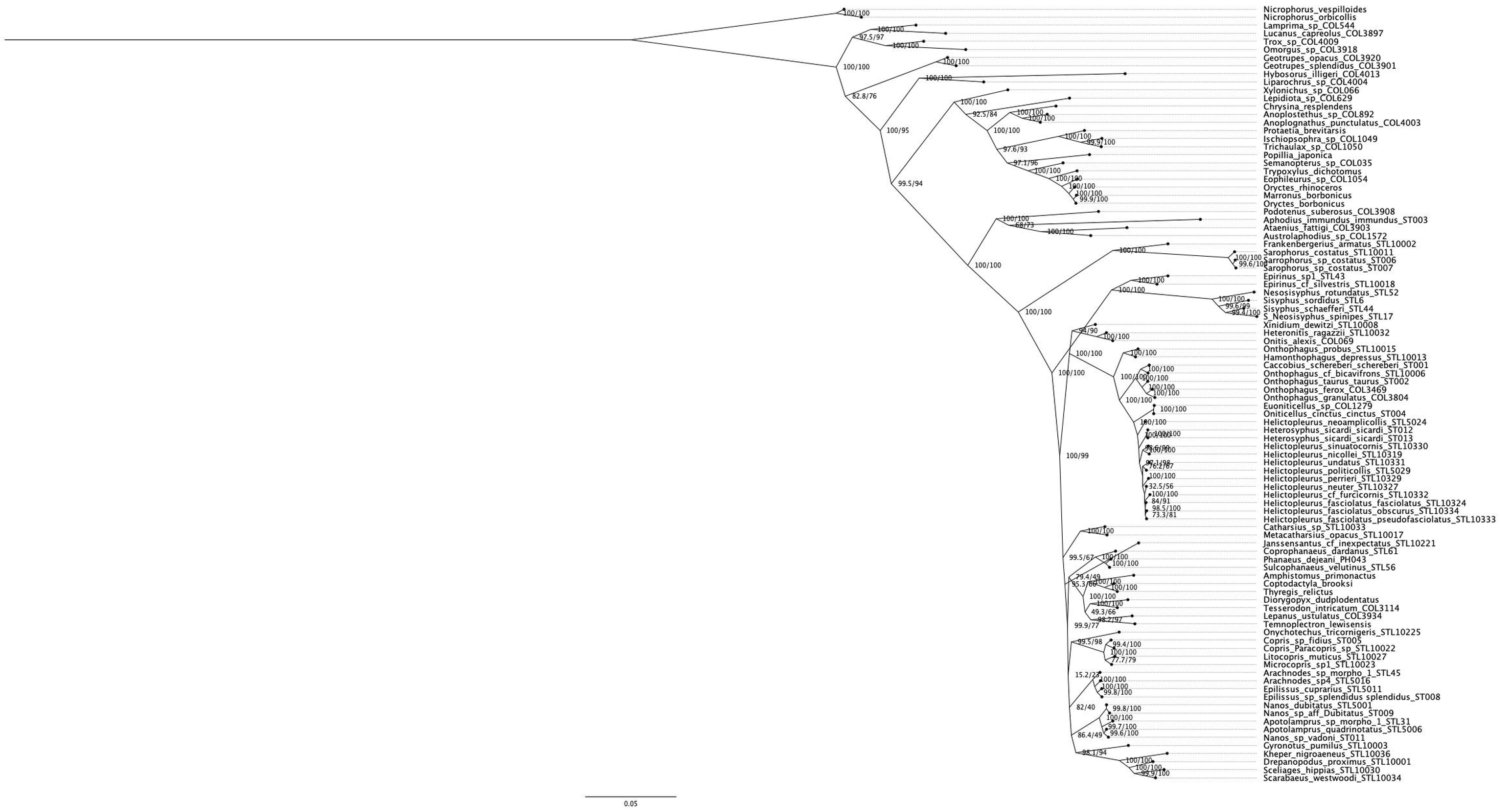

Supplement: S3 Fig — Phylogeny reconstructed with 70p dataset containing all taxa assessed in this study and bootstrap/SH values. (TIF) [file pone.0309596.s009.tif]

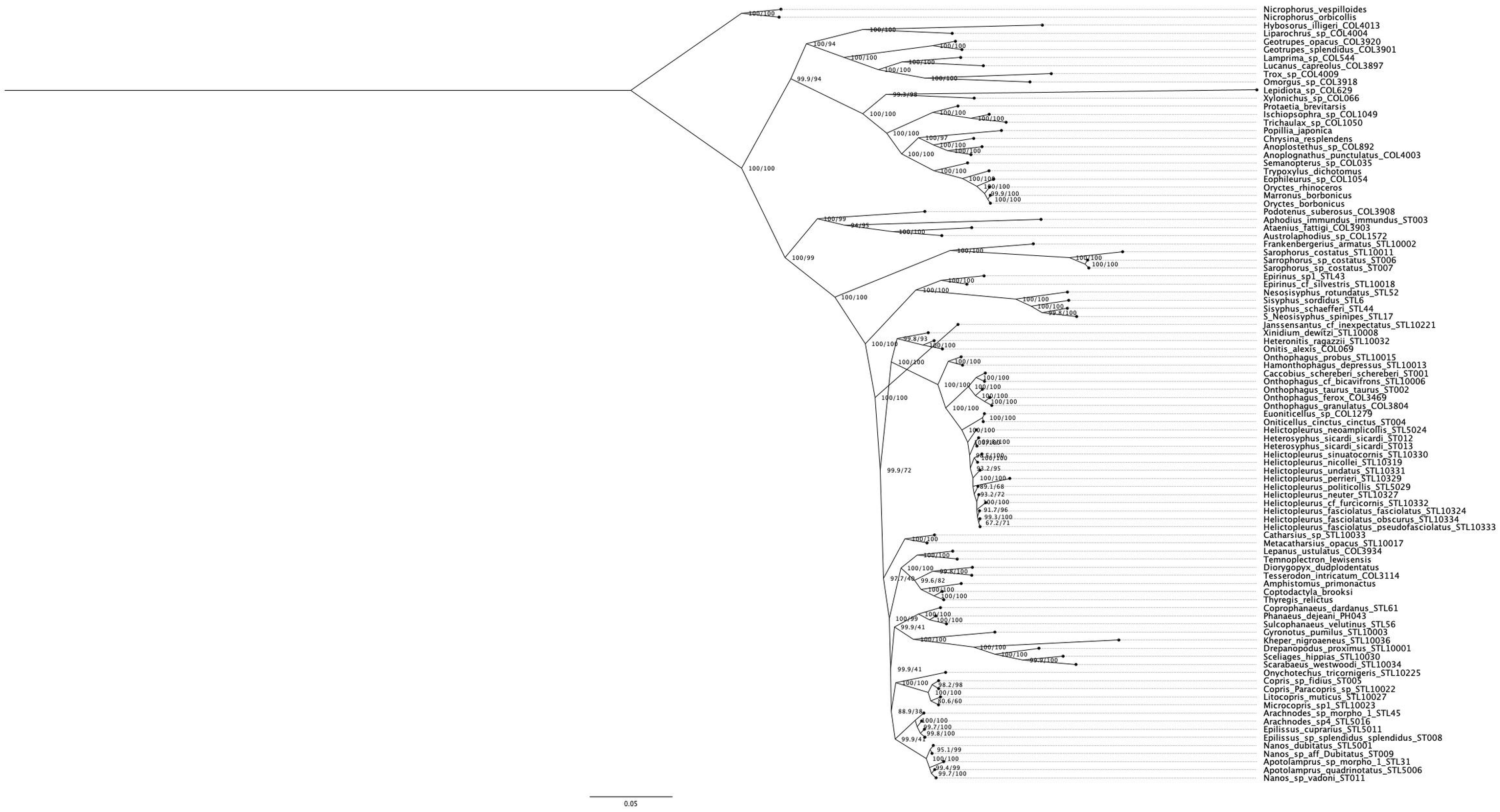

Supplement: S4 Fig — Phylogeny reconstructed with 70p dataset containing all taxa assessed in this study and bootstrap/SH values. (TIF) [file pone.0309596.s010.tif]

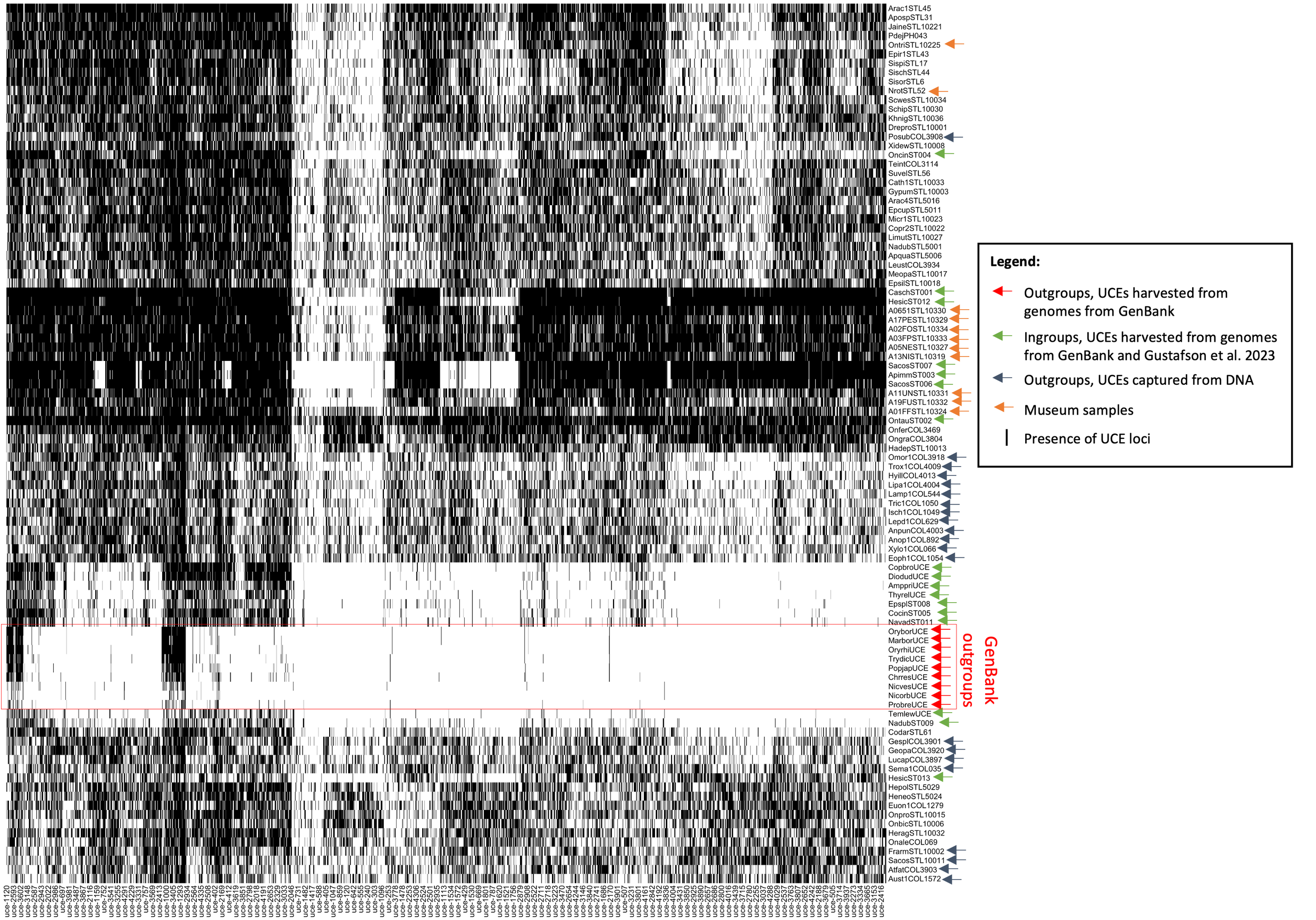

Supplement: S5 Fig — The absence is shown in white. The amount of missing data is highlighted with red arrows and rectangles for UCEs captured from genomes from Genbank. (TIF) [file pone.0309596.s011.tif]
